# Defending Behavior and Victimization: Between- and Within-Person Associations

**DOI:** 10.1007/s10964-025-02168-x

**Published:** 2025-03-20

**Authors:** Sarah T. Malamut, Claire F. Garandeau, Christina Salmivalli

**Affiliations:** INVEST Research Flagship, Department of Psychology, University of Turku, Turku, Finland

**Keywords:** Defending, Victimization, Longitudinal, Within-person associations

## Abstract

Anti-bullying interventions often encourage peer bystanders to defend their victimized peers. However, concerns have been raised that defending could put youth at risk for being victimized themselves. Despite these concerns, there is limited research on the longitudinal links between defending and victimization. Addressing limitations of previous research, the current study examined bidirectional associations between three types of peer-reported defending (comforting defending, assertive defending, reporting to authority) and (self- and peer-reported) victimization, teasing apart between- and within-person associations using random-intercept cross-lagged panel models. Participants included 5123 Finnish adolescents (45.9% self-identified as a boy; T1 *M*_age_ = 13.06, *SD* = 1.69, 93.5% born in Finland) in grades 4 to 9. There was a significant, negative between-person association only between comforting defending and self-reported victimization. There were no significant prospective within-person associations between any type of defending and self- or peer-reported victimization. The findings did not indicate that defending was a risk factor for subsequent victimization.

## Introduction

Many anti-bullying interventions consider ways to encourage peer bystanders to defend their victimized peers (Gaffney et al., 2021), and defending does appear to be associated with reduced levels of bullying in the classroom (Nocentini et al., 2013). Defending others, unlike many other forms of prosocial behavior, inherently involves some degree of risk (potential negative feedback from peers and/or retaliation from the perpetrator). As defending peers is a critical component of many anti-bullying interventions (Gaffney et al., 2021), it is crucial to examine whether encouraging this behavior could inadvertently put youth at risk for subsequent victimization. However, it is still unknown whether defending actually increases the likelihood that youth are victimized and whether this is the case for all types of defending. It is also important to consider the other direction - that victimized youth are more likely to defend their fellow victimized peers over time. The current study aims to clarify the bidirectional associations between defending and victimization, using four waves of data. This study goes beyond past research on this topic by differentiating between- and within-person associations (using random-intercept cross-lagged panel modeling: RI-CLPM: Hamaker et al., 2015), while separately considering three different types of peer-reported defending (i.e., assertive defending, comforting defending, reporting to authorities) and considering multiple informants of victimization (self and peers).

### Bidirectional Associations Between Different Types of Defending and Victimization

In line with theoretical models of Prosocial Risk Taking (Do et al., 2017), defending is a voluntary behavior that is enacted to benefit someone else but also includes an unknown risk – thus involving both prosociality and risk-taking. One key risk for defenders is the potential to be judged negatively by peers or become the next target, which may be particularly salient in adolescence when social relationships with peers are especially important (Gavin & Furman, 1989). Although youth themselves note that a fear of victimization may preclude them from defending their peers (Strindberg et al., 2020), the limited longitudinal research has produced mixed findings regarding whether defending is (Huitsing et al., 2014) or is not (Malamut et al., 2023; Meter & Card, 2015) actually positively associated with victimization over time.

However, a crucial limitation of previous studies is that they have studied the links between defending and victimization from a between-person perspective. Although this provides information regarding whether those who defend (or are victimized) more than their peers on average also tend to be victimized (or defend) more than their peers on average over time, between-person associations do not answer whether the act of defending itself impacts youth’s subsequent risk for becoming more victimized. To answer this critical question (whether defending puts youth at risk for victimization), as well as whether victimization experiences may increase youth’s likelihood to defend in the future, it is necessary to also examine within-person changes. By disentangling between- and within-person associations, it is possible to investigate whether youth’s own defending or victimization experiences are related to that individual’s subsequent victimization or defending. Specifically, RI-CLPMs test whether youth who score higher than expected on defending or victimization (compared to their usual levels) is related to scoring higher than expected on victimization or defending (respectively) at the next time point.

As posited in a model of defending behavior as an act of moral courage (Pouwels et al., 2019 – adapted from Latané & Darley, 1970), the final stage before enacting defending behavior is a cost-benefit analysis (i.e., whether the costs of not intervening are greater than the costs of intervening). Although most previous research examining the longitudinal link between defending and victimization did not differentiate between different types of defending behaviors (see Lambe & Craig, 2022 and Malamut et al., 2023 as exceptions), different strategies of defending may not be equally risky in terms of potential future victimization. Recent research distinguishes three main types of defending behaviors (sometimes complemented by additional ones): directly confronting the perpetrator (assertive defending), comforting the victimized peer, and reporting the bullying to an authority (e.g., Lambe & Craig, 2020; Yun, 2020). The extent to which defending could be a risk factor for future victimization may differ depending on how the defending is enacted.

In particular, risky defending strategies may entail those that overtly go against the perpetrator (e.g., assertive defending). Perpetrators tend to be popular (Wiertsema et al., 2023) and influential in the peer group (Sandstrom, 2011), with access to social resources. As such, directly confronting perpetrators may put defenders at odds with powerful classmates, and at risk for retaliation. Although it has been argued that directly confronting the perpetrator is a risky form of defending, some previous research did not find a prospective link between direct defending and victimization (Malamut et al., 2023). Still, this study only focused on between-person associations, and did not control for the other temporal direction. Thus, it is still unclear whether assertive defending is related to within-person changes in victimization. On the within-person level, the hypothesis that higher levels of engaging in assertive defending (compared to one’s usual levels) is associated with increases in victimization (compared to one’s usual levels) will be tested.

Other than directly confronting the perpetrator, youth may choose to report the bullying to an authority. Although this strategy avoids confronting the perpetrator, it may still lead to backlash from the bully or other peers. Youth have disclosed being hesitant to report bullying to authorities out of fear of being labeled a “snitch” and subsequently being targeted (Forsberg et al., 2018). Youth also report believing that disclosing bullying to authorities would signal a lack of autonomy (needing to get an adult involved rather than handling it themselves; Boulton et al., 2017). In support of these concerns, a recent study found that high levels of reporting bullying to authorities was indeed associated with high levels of victimization over time (Lambe & Craig, 2022). Therefore, individuals who report to authority are expected to experience within-person increases in victimization over time (relative to their usual levels).

Whereas only a few studies have examined the longitudinal associations between defending and victimization, even fewer have examined both temporal directions in the same study (i.e., both defending predicting victimization *and* victimization predicting defending; Huitsing et al., 2014; Lambe & Craig, 2022). Despite being vulnerable themselves, victimized youth may want to defend other victimized peers as they can relate to their distress. Previous research has indeed found positive longitudinal associations between victimization and relative within-person increases in (cognitive) empathy for victimized peers (Trach et al., 2023), and some evidence has been found that victimized youth who share a bully are more likely to defend each other over time (Huitsing et al., 2014).

As the potential costs of defending are particularly relevant for vulnerable youth, victimized youth may be likely to choose defending strategies that avoid direct confrontation with the perpetrator, such as comforting the victim and involving an authority. The only study to have examined whether victimization is prospectively associated with different types of defending strategies did find that youth with high levels of victimization engaged in high levels of reporting the bullying, but surprisingly did not find a significant association between victimization and comforting (Lambe & Craig, 2022). Given the dearth of research on this topic, additional research is needed; particularly regarding within-person changes. Victimization is expected to be linked to within-person increases in comforting the victim and reporting the bullying to an authority (relative to their usual levels).

### Informant of Victimization

When examining the links between defending and victimization, some studies have used only self-perceived victimization (either using a self-report questionnaire: Lambe & Craig, 2022, or asking participants to report who targets them: Huitsing et al., 2014), some have used only peer-reported victimization (Meter & Card, 2015), and others have used both self- and peer-reported victimization (Malamut et al., 2023). Considering multiple informants of victimization experiences is crucial, as distinct profiles (both in terms of behaviors and adjustment) have been found depending on the informant of victimization (e.g., Bouman et al., 2012). Indeed, the correspondence between self- and peer-reported victimization tends to be relatively small (e.g., Malamut et al., 2021). Previous research recommends considering both informants as each measure could identify victimized youth that would not be captured by a single informant (e.g., Oldenburg et al., 2015). Specifically, self-reported victimization can capture victimization that peers may be unaware of, and peer-reported victimization can capture victimization that participants may be hesitant to disclose (Graham & Juvonen, 1998). Thus, in the current study, both self- and peer-reports of victimization are used.

### Gender and Grade Level Differences

As exploratory analyses, potential gender and grade level differences will be considered in all models. Although previous studies did not find any gender differences in the link between defending and victimization (Lambe & Craig, 2022), research has found gender differences in the extent to which boys and girls engage in different defending strategies (e.g., Wang et al., 2023). For example, one study has found that boys were more likely to engage in assertive defending whereas girls were more likely to engage in comforting defending, with no gender differences for reporting to authority (Wang et al., 2023). In terms of potential grade level differences, previous studies have primarily focused on only early to mid-adolescents (approximately ages 11–16: Lambe & Craig, 2022; Malamut et al., 2023; Meter & Card, 2015), or only children (approximately ages 8–11: Huitsing et al., 2014).

Guided by the theoretical model of Prosocial Risk Taking (Do et al., 2017), adolescents (compared to younger children) may be more likely to adapt their willingness to engage in different types of defending based on their perception of peers’ reactions, as they are particularly sensitive to social evaluation. In addition, bullies tend to be more popular in adolescence than childhood (Pouwels et al., 2018), which could have implications both for the potential consequences of defending victimized peers, as well as the likelihood of whether victimized youth are willing to defend others. Thus, the current study will examine these questions in youth in grades 4 to 9 (approximately 10–16 years old) and will explore whether the link between different types of defending and victimization differ between primary school students (grades 4–6) and secondary school students (grades 7–9).

## Current Study

Defending is considered a risky prosocial behavior, and youth often report being hesitant to defend their peers out of fear of becoming victimized themselves. However, it is not yet clear whether defending itself actually puts youth at risk for subsequent victimization (e.g., within-person increases). The current study aims to further elucidate the prospective links between defending and victimization, by addressing several limitations of existing research. Specifically, this study examines the bidirectional between- and within-person associations of three different types of defending behavior and victimization (using both self- and peer-reported victimization). To do so, random-intercept cross-lagged panel models (RICLPMs) are used, which allow for the investigation of relative within-person changes. Although prior longitudinal studies did not find a (between-person) positive association between direct forms of defending and victimization, assertive defending is hypothesized to be associated with within-person increases in victimization, given conceptual rationales for such a link. In addition, reporting bullying to an authority is hypothesized to be linked to within-person increases in victimization. No association between comforting defending and subsequent within-person increases in victimization is hypothesized. However, victimization is expected to be linked to within-person increases in comforting defending, as victimized youth are aware first-hand of the pain of being victimized. Likewise, victimization is expected to be linked to within-person increases in reporting bullying to authorities. No association between victimization and within-person increases in assertive defending is hypothesized. Although there are no a priori hypotheses for differences when predicting self- vs. peer-reported victimization, both informants are considered given known differences in the correlates of victimization between informants. In addition, exploratory analyses will be conducted to consider possible gender and grade level differences.

## Method

### Participants and Procedure

The data for this study included Finnish adolescents in grades 4 to 9. Data collection occurred in seven waves over the course of three academic years (Wave 1 – October 2020, Wave 2 – February 2021, Wave 3 – May 2021, Wave 4 – October 2021, Wave 5 – February 2022, Wave 6 – May 2022, Wave 7 – February 2023). The current study utilizes data from Wave 1, Wave 3, Wave 4 and Wave 6 (henceforth referred to as T1, T2, T3 and T4, respectively), as these waves included measures regarding youth’s defending behaviors. Active parental consent was obtained from 68% of the target sample at T1.

The final sample included 5123 students (45.9% self-identified as a boy; T1 *M*_age_ = 13.06, *SD* = 1.69, 93.5% born in Finland) in grades 4 to 9 (42.8% primary school grades 4–6, 57.2% secondary school grades 7–9 at T1) who participated in at least 1 wave. Missing data was addressed using full information maximum likelihood estimation (FIML). Proportion scores (see Measures) were only calculated for classrooms with at least 10 participating students and with participation rates greater or equal to 40% at a given wave, to increase the reliability of peer nomination items (Cillessen & Marks, 2011).

Online questionnaires were administered to the participants under the supervision of classroom teachers, who were given detailed instructions about the data-collection. Students were informed that their answers were confidential, and that they could opt out of the study at any time. The study was approved by the Ethical Board of the University of Turku. The hypotheses and analytic plan for main analyses were preregistered online at OSF (https://osf.io/4a2jk).

### Measures

#### Defending

Defending was assessed using peer nominations; students were asked “who in your class acts like this in bullying situations?”. Three types of defending behaviors were assessed: assertive defending (2 items: “tries to make others stop the bullying”, “gets angry at the bully/ies”), comforting defending (2 items: “consoles the victim afterwards”, “is friendly towards the victim”), and reporting the bullying to authorities (1 item: “reports the bullying to an adult”). For each item, participants could nominate an unlimited number of classmates; they could also choose “nobody” or skip the question. For each student, the number of received nominations was divided by the number of potential nominators for each item, resulting in five separate proportion scores. The scores for assertive defending (two items, *rs* >= 0.71) were averaged, as were the scores for comforting defending (two items, *rs* > = 0.86).

#### Victimization

Peer-reported victimization was assessed with three peer-nomination items from the Participant Role Questionnaire (PRQ; Salmivalli & Voeten, 2004; i.e., “s/he is called names and made fun of”, “s/he is pushed and hit”, “s/he is usually talked about with a bad tone”) were used to assess victimization. For each item, participants could nominate an unlimited number of classmates. Proportion scores were computed for each participant by dividing the sum of received nominations by the number of possible nominators within each class to form a proportion score for each participant. The final victimization score was created by averaging the proportion scores across the 3 items for each student, with scores ranging from 0 to 1 (reliability of ωs > = 0.76 at each wave).

Self-reported victimization was assessed using 10 items of the revised Olweus Bully/Victim questionnaire (Olweus, 1996). Participants reported how frequently they experienced different forms of victimization (e.g., “I was hit, kicked, or pushed”, “I was called nasty names or laughed in my face or hurt by insults”), using a 5-point scale (0 = *not at all*, 4 = *several times a week*). A total self-reported victimization score was calculated by averaging participants’ responses on the 10 items (reliability of ωs > = 0.78 at each wave).

#### Gender

Participants reported their gender (1 = “girl”, 2 = “boy”, 3 = “I don’t feel like either a girl or a boy”, 4 = “I don’t want to answer”). Gender was then dummy coded as 0 = *boy*, 1 = *girl*, with values of students who chose “3” or “4” (neither or choose not to answer) set as missing due to their small percentage (3.5%, 5.0%, 5.5%, 8.1% at T1 to T4).

#### Grade Level

Participants’ grade level was dummy coded as 0 = *primary school* (grades 4–6), 1 = *secondary school* (grades 7–9).

### Analytic Plan

Random-intercept cross-lagged panel models (RI-CLPM) were conducted to examine the bidirectional associations between defending and victimization. In contrast to the traditional CLPM, which assumes that all individuals vary around a common group mean, the RI-CLPM assumes that there are stable between-person differences. The RI-CLPM differentiates this between-person variance, which represents the part that remains stable over time for individuals, from the within-person variance, which represents the difference between the observed scores and the expected mean score for each individual (i.e., person-mean centered scores, captured with a latent factor at each wave).

At the between-person level, random intercepts were added that reflect the trait-like differences between individuals in defending and victimization, based on the observed scores of each variable at each time point, with all factor loadings constrained to 1. Stable between-person relations among the study variables were controlled for by adding covariances between the random intercepts. At the within-person level, the observed score for each variable at each time point was loaded on its own latent factor (one latent factor for each variable) with the factor loadings constrained to 1. These latent factors represent each individual’s expected mean score on the variable, that is, their expected score given both their own stable trait factor and the sample mean (see Hamaker et al., 2015, pp. 104–105 for more information). The RI-CLPMs were conducted using the lavaan package in R (Rosseel, 2012). In each model, autoregressive paths, cross-lagged paths, covariances at T1, and covariances between residuals at T2, T3 and T4 were included between the within-person latent factors. The cross-lagged paths between defending and victimization were tested separately for the three types of defending, such that each model only included cross-lagged paths between victimization and one type of defending at a time. Although the preregistration included controlling for the random intercepts for the other two defending variables (covariances between the random intercepts), this proved too complex for the models to converge.

Gender and school level (i.e., primary or secondary school) were also controlled on the observed variables of defending and victimization at each wave. The models were estimated using full information maximum likelihood estimation with robust standard errors (MLR) to account for missing data and nonnormality. Two participants were missing data on all relevant variables, resulting in the inclusion of 5121 participants in the RI-CLPMs. Model fit was evaluated based on conventional measures: CFI > 0.9, TLI > 0.9, RMSEA < 0.08, and SRMR close to zero (Kline, 2004). Potential dependencies in the data due to students being nested within classrooms were corrected using the “cluster” option in lavaan (using classroom at T1). As an exploratory analysis, gender and grade level were also tested as moderators, by conducting multigroup analyses. For the analyses testing grade level as a moderator, participants who changed grade levels between T2 and T3 were excluded. Using the Satorra Bentler scaled chi-square difference test (Satorra & Bentler, 1994), a model in which all parameters were constrained to be equal (constrained model) with a model in which all parameters were freely estimated (unconstrained model) were compared. In case the unconstrained model fits the data significantly better than the unconstrained model, the beta estimates of this unconstrained model and their 95% CIs for the cross-lagged effects were compared for boys and girls, and for primary and secondary students. Differences between beta estimates were considered significant only when the 95% CIs of the beta estimates of one group did not include the beta estimates for the other group and vice versa (Pfister & Janczyk, 2013). Separate models were conducted for self- and peer-reported victimization.

## Results

Descriptive statistics and correlations are presented in Table 1. Due to the large sample size, correlations are considered significant at *p* < 0.001. The three defending types were strongly, positively correlated with each other at each wave. There were small to moderate correlations between self- and peer-reported victimization at each wave. Neither self- nor peer-reported victimization were significantly correlated with any defending type at any wave.

**Table 1:** Descriptive statistics and correlations

	M (SD)	1	2	3	4	5	6	7	8	9	10	11	12	13	14	15	16	17	18	19
1. SR Vic T1	1.21 (0.45)	–																		
2. SR Vic T2	1.23 (0.48)	0.47^***^	–																	
3. SR Vic T3	1.20 (0.47)	0.34^***^	0.56^***^	–																
4. SR Vic T4	1.23 (0.52)	0.30^***^	0.35^***^	0.46^***^	–															
5. PR Vic T1	0.02 (0.04)	0.29^***^	0.18^***^	0.09^***^	0.15^***^	–														
6. PR Vic T2	0.02 (0.05)	0.28^***^	0.23^***^	0.10^***^	0.11^***^	0.65^***^	–													
7. PR Vic T3	0.02 (0.04)	0.23^***^	0.17^***^	0.15^***^	0.17^***^	0.53^***^	0.65^***^	–												
8. PR Vic T4	0.02 (0.04)	0.31^***^	0.18^***^	0.06	0.20^***^	0.49^***^	0.58^***^	0.65^***^	–											
9. Comf. T1	0.21 (0.18)	−0.00	0.01	−0.04	−0.05	−0.01	−0.06	−0.03	−0.05	–										
10. Comf. T2	0.19 (0.17)	−0.03	0.01	−0.00	−0.03	−0.03	−0.04	−0.00	−0.03	0.77^***^	–									
11. Comf. T3	0.19 (0.18)	−0.02	−0.00	−0.02	−0.05	−0.02	−0.06	0.00	−0.04	0.69^***^	0.77^***^	–								
12. Comf. T4	0.14 (0.16)	−0.04	0.01	−0.03	−0.03	−0.04	−0.06	−0.04	−0.04	0.64^***^	0.67^***^	0.75^***^	–							
13. Assert. T1	0.13 (0.13)	0.02	0.03	−0.02	−0.03	0.02	−0.02	0.02	−0.00	0.86^***^	0.71^***^	0.66^***^	0.64^***^	–						
14. Assert. T2	0.11 (0.12)	−0.00	0.04	0.02	−0.02	−0.01	0.00	−0.04	0.00	0.69^***^	0.86^***^	0.72^***^	0.66^***^	0.70^***^	–					
15. Assert. T3	0.11 (0.12)	−0.00	−0.00	−0.02	−0.04	−0.01	−0.01	0.03	−0.03	0.61^***^	0.69^***^	0.87^***^	0.73^***^	0.66^***^	0.73^***^	–				
16. Assert. T4	0.09 (0.12)	−0.04	−0.00	−0.04	−0.02	−0.03	−0.03	0.01	−0.01	0.57^***^	0.60^***^	0.68^***^	0.91^***^	0.62^***^	0.66^***^	0.74^***^	–			
17. Report. T1	0.19 (0.21)	0.04	0.03	−0.03	−0.04	0.05	−0.01	0.06	0.02	0.82^***^	0.71^***^	0.66^***^	0.61^***^	0.82^***^	0.70^***^	0.62^***^	0.54^***^	–		
18. Report. T2	0.15 (0.17)	0.02	0.03	0.02	−0.02	0.03	0.00	0.02	0.02	0.66^***^	0.79^***^	0.77^***^	0.68^***^	0.64^***^	0.83^***^	0.71^***^	0.61^***^	0.74^***^	–	
19. Report. T3	0.15 (0.17)	0.03	0.00	0.01	−0.02	0.05	0.03	0.05	0.00	0.59^***^	0.68^***^	0.84^***^	0.73^***^	0.59^***^	0.69^***^	0.84^***^	0.67^***^	0.66^***^	0.79^***^	–
20. Report. T4	0.11 (0.16)	−0.01	−0.00	−0.03	−0.01	0.01	0.00	0.03	0.03	0.59^***^	0.58^***^	0.70^***^	0.87^***^	0.60^***^	0.63^***^	0.72^***^	0.87^***^	0.61^***^	0.68^***^	0.73^***^

*SR Vic* self-reported victimization, *PR* peer-reported victimization, *Comf*. comforting defending, *Assert*. assertive defending, *Report*. reporting to authority

Due to the large sample size, correlations were considered significant at *p* < 0.001

^***^p < 0.001

### Random-Intercept Cross-lagged Panel Models

To determine the amount of variance that could be explained by stable differences between persons, intraclass correlations (ICCs) were calculated for each variable (comforting defending, assertive defending, reporting to authority, peer-reported victimization, and self-reported victimization) across the four waves (e.g., Mulder, 2023). The ICCs for comforting defending, assertive defending and reporting to authority were 69.5%, 67.8% and 64.3%, respectively, indicating that approximately 60–70% of the variance in each defending type could be explained by between-person differences. For peer- and self-reported victimization, 59.3% and 35.1% of the variance, respectively, could be explained by stable differences between persons.

### Comforting Defending and Victimization

**Fig. 1:**
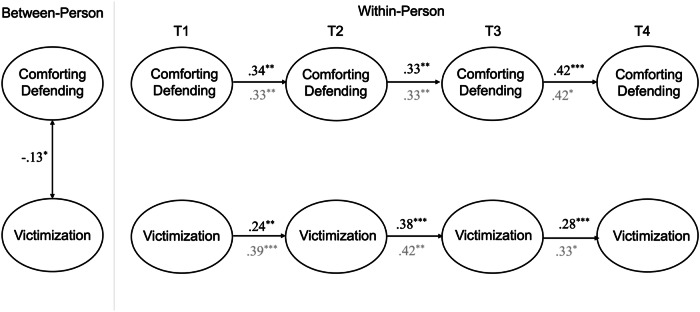
Simplified random-intercept cross-lagged panel model for comforting defending and victimization. Standardized coefficients are presented. Coefficients from the model with self-reported victimization are presented in black whereas the coefficients from the model with peer-reported victimization are presented in gray. *p < 0.05, **p < 0.01, ***p < 0.001

The RI-CLPM examining the links between comforting defending and self-reported victimization had adequate fit *χ2*(44) = 3520.43, *p* < 0.001, CFI = 1.00, TLI = 1.00, RMSEA = 0.00 (90% CI [0.00, 0.02]), SRMR = 0.01, as did the model testing links between comforting defending and peer-reported victimization, *χ2*(44) = 3235.03, *p* < 0.001, CFI = 1.00, TLI = 0.99, RMSEA = 0.04 (90% CI [0.00, 0.08]), SRMR = 0.02. Separate models were tested for peer- and self-reported victimization, but the standardized estimates for both models are presented in Fig. 1.

As shown in Fig. 1, there was a significant, but negative, between-person association linking comforting defending and self-reported victimization. This indicates that youth who tended to score higher on comforting defending across all waves also tended to report lower levels of victimization across measurement waves, compared to individuals with lower levels of comforting defending. There was no significant between- person association for comforting defending and peer-reported victimization.

At the within-person level, there were no significant concurrent or prospective associations between comforting defending and (self- or peer-reported) victimization. There was, however, consistent within-person carry over effects for victimization (regardless of informant) and comforting defending. This indicates that, on average, individuals who scored higher than their expected score tended to score above their expected score again at the following time point.

### Assertive Defending and Victimization

**Fig. 2:**
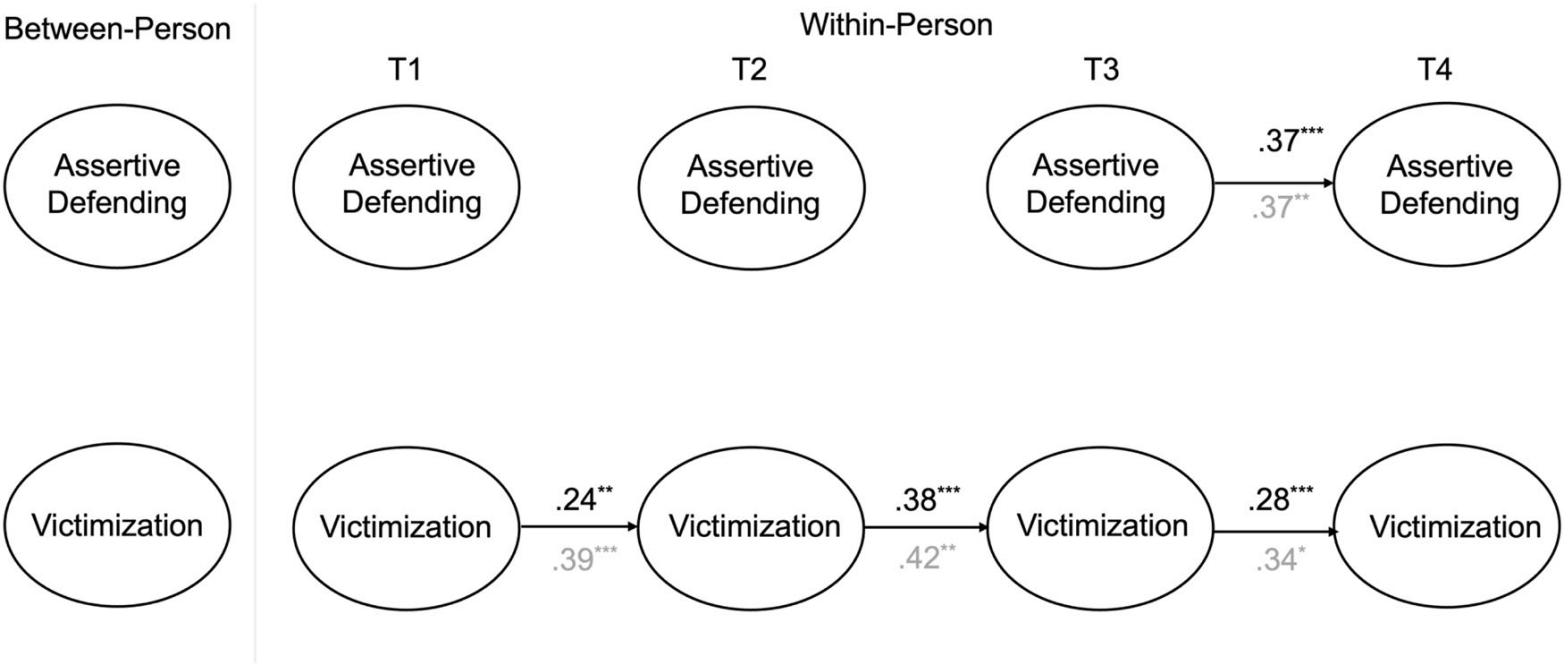
Simplified random-intercept cross-lagged panel model for assertive defending and victimization. Standardized coefficients are presented. Coefficients from the model with self-reported victimization are presented in black whereas the coefficients from the model with peer-reported victimization are presented in gray. *p < 0.05, **p < 0.01, ***p < 0.001

The RI-CLPM examining the links between assertive defending and self-reported victimization had adequate fit *χ2*(44) = 2323.44, *p* < 0.001, CFI = 1.00, TLI = 1.00, RMSEA = 0.00 (90% CI [0.00, 0.04]), SRMR = 0.01, as did the model testing assertive defending and peer-reported victimization, *χ2*(44) = 2263.01, *p* < 0.001, CFI = 1.00, TLI = 0.99, RMSEA = 0.03 (90% CI [0.00, 0.08]), SRMR = 0.02. As before, separate models were tested for peer- and self-reported victimization (see Fig. 2).

The between-person associations for assertive defending and self-reported victimization, as well as for assertive defending and peer-reported victimization, were both nonsignificant.

At the within-person level, there were no significant concurrent or prospective associations between assertive defending and (self- or peer-reported) victimization. There were consistent within-person carry over effects for both self- and peer-reported victimization. On average, individuals who scored above their expected score on victimization at a given time point tended to still score above their expected score at the following time point. However, there was only a significant within-person carry over effect for assertive defending from T3 to T4.

### Reporting to Authority and Victimization

**Fig. 3:**
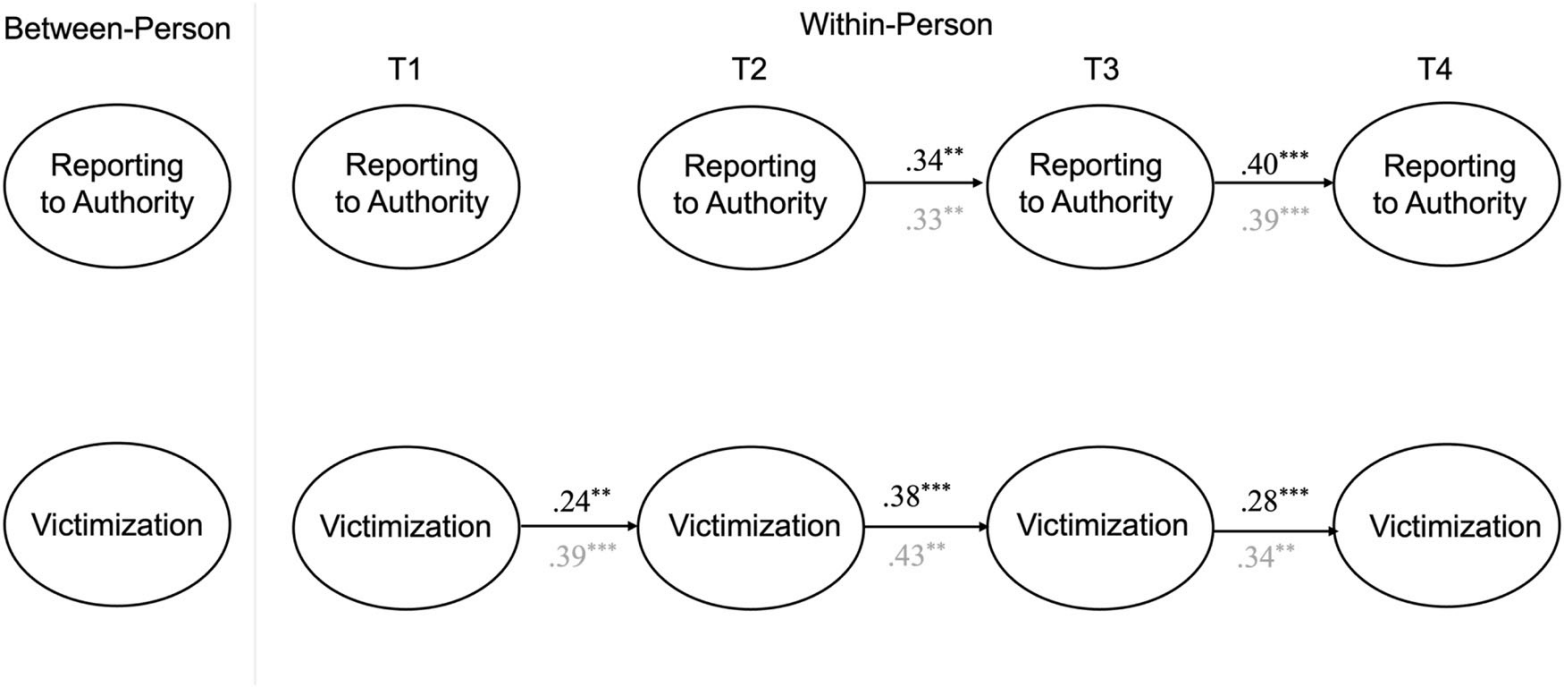
Simplified random-intercept cross-lagged panel model for reporting to authority and victimization. Standardized coefficients are presented. Coefficients from the model with self-reported victimization are presented in black whereas the coefficients from the model with peer-reported victimization are presented in gray. **p < 0.01, ***p < 0.001

The RI-CLPM examining the links between reporting to authority and self-reported victimization had adequate fit *χ2*(44) = 2946.03, *p* < 0.001, CFI = 1.00, TLI = 1.00, RMSEA = 0.00 (90% CI [0.00, 0.02]), SRMR = 0.01, as did the model testing reporting to authority and peer-reported victimization, *χ2*(44) = 2789.04, *p* < 0.001, CFI = 1.00, TLI = 0.99, RMSEA = 0.03 (90% CI [0.00, 0.08]), SRMR = 0.02. As before, separate models were tested for peer- and self-reported victimization (see Fig. 3).

The between-person associations for reporting to authority and self-reported victimization, as well as for reporting to authority and peer-reported victimization, were both nonsignificant.

At the within-person level, there were no significant concurrent or prospective associations between reporting to authority and (self- or peer-reported) victimization. There were consistent within-person carry over effects for both self- and peer-reported victimization. On average, individuals who scored above their expected score on victimization at a given time point tended to score above their expected score again at the following time point. With the exception of T1 to T2, there were also significant within-person carry over effects for reporting to authority.

### Gender and Grade Level as Moderators

As preregistered exploratory analyses, possible gender or grade level differences were also considered. For each of the six tested models described above (comforting defending & self-reported victimization, comforting defending & peer-reported victimization, assertive defending & self-reported victimization, assertive defending & peer-reported victimization, reporting to authority & self-reported victimization, reporting to authority & peer-reported victimization), multigroup analyses were first conducted to test for gender moderation (with grade level still controlled for). No significant gender differences were found in any model (*p*s > = 0.37).

Next, multigroup analyses were conducted to test for grade level moderation (with gender still controlled for). There was no significant grade level difference (primary vs. secondary school grades) in the model testing comforting defending and peer-reported victimization (*p* = 0.57). When testing comforting defending and self-reported victimization, the unconstrained model fit better (*p* < 0.001). There, however, were no significant cross-lagged paths between comforting defending and self-reported victimization for either level of schooling (see Supplemental Table S1). There were significant grade level differences for the models testing assertive defending (*ps* = <0.01) and reporting to authority (*ps* = < 0.003). For the models testing assertive defending, the paths that differed between the two groups (when comparing the 95% CIs) *and* reached significance for either group primarily pertained to the autoregressive paths for assertive defending. There were significant within-person carry over effects for assertive defending in primary school, but not secondary school – this pattern was consistent in both the model with assertive defending and self-reported victimization (see Supplemental Table S2) as well as in the model with assertive defending and peer-reported victimization (see Supplemental Table S3). There was a small, positive within-person association between assertive defending and self-reported victimization at T1 for secondary students only (see Supplemental Table S2).

Lastly, grade level differences were explored when testing the models between reporting to authority and victimization (see Supplemental Tables S4 & S5). Significant within-person carry over effects were found for reporting to authority in primary school, but not in secondary school. There was a positive within-person association between reporting to authority and peer-reported victimization at T2 for secondary students only (see Supplemental Table S5). Across all models, there were no cross-lagged associations that differed between the two groups (when comparing the 95% CIs) *and* reached significance for either group.

### Sensitivity Analyses

Given that other variables, such as popularity or individuals’ own bullying behavior, may be related both to the likelihood of defending as well as any potential social consequences, a series of sensitivity analyses were conducted while controlling for peer-reported popularity and peer-reported bullying behavior. To control for these variables, additional random intercepts were added for popularity and bullying (with the procedure described above in the analytic plan), with the covariances between all random intercepts included. As with defending and victimization, within-person latent factors were created for popularity and bullying. In each sensitivity analysis, autoregressive paths, cross-lagged paths, covariances at T1, and covariances between residuals at T2, T3 and T4 were included between the within-person latent factors, controlling for grade level and gender. The sensitivity analyses were conducted for each type of defending (comforting, assertive, reporting to authority) and each informant of victimization (self, peer), resulting in six additional models. Across all six models controlling for popularity and bullying, there was only one significant cross-lagged path between defending and victimization: reporting to authority at T2 was negatively associated with peer-reported victimization at T3 (*p* = 0.045).

## Discussion

Although peer bystanders can play an important role in bullying (Salmivalli et al., 2021), concerns have been raised regarding whether defending victimized peers may put defenders at risk themselves, especially as it is a fear shared by many students (Strindberg et al., 2020). Despite these concerns, only a few studies have actually examined the longitudinal links between defending and victimization, with inconsistent findings. The current study aimed to clarify the longitudinal, bidirectional associations between defending and victimization, while addressing key limitations of previous research. Specifically, this study disentangled between- and within-person associations, while considering both temporal directions (defending predicting victimization, victimization predicting defending), multiple types of defending, and two informants of victimization (self- and peer-reported).

### Between-Person Associations of Defending and Victimization

Whereas previous research has found some evidence of a positive concurrent, between-person association between defending and victimization (Ma et al., 2019), no evidence of such a positive link was found in the current study. There was no significant between-person association among either assertive defending or reporting to authority and victimization (whether self- or peer-reported). In contrast, there was a significant, *negative* between-person association for comforting defending and self-reported victimization (but not peer-reported victimization). Given that it is a between-person association aggregating across all measurement waves, directionality cannot be teased apart. Yet, it suggests that adolescents who self-reported high levels of victimization (across all waves) were less likely to be seen by peers as engaging in comforting defending (or that those who engaged in comforting defending more than others tended to report being victimized less frequently than others). This result was unexpected, especially given that victimized youth tend to be friends with other victimized youth (e.g., Sentse et al., 2013), and presumably friends would be likely to comfort one another. However, it is possible that adolescents who feel more vulnerable compared to their peers (i.e., higher in self-reported victimization) may be inclined to try to distance themselves from their victimized classmates to protect themselves from further victimization.

These findings are not wholly consistent with previous studies examining concurrent, between-person associations between defending and victimization. A meta-analytic review found a positive, albeit small, concurrent association between defending and victimization (Ma et al., 2019). Yet, the meta-analysis did note substantial variation in the effect sizes found across studies (with 39 reporting a positive effect size, but 21 reporting a negative effect). Moreover, significant moderation was found such that a positive association was found when defending was self-reported, but not when it was peer-reported, among youth older than nine years old. Peer-reported defending, however, provides the benefit of a more objective reporter(s) of behavior (i.e., less susceptible to social desirability bias or reporting idealized self-behavior rather than actual behavior). In addition, peer-reported defending may be more relevant for the question of whether defending confers risk for individuals – as peers presumably must be aware that defending has occurred in order for defending to be risky or lead to retaliation.

### Within-Person Associations of Defending and Victimization

On the within-person level, there were consistent within-person carry-over effects for victimization (regardless of informant). This suggests that even after accounting for stable, between-person differences in victimization experiences, there are also lingering effects of being victimized that carry over from one wave to the next. Consistent within-person carry-over effects were also found for comforting defending. However, the findings for assertive defending and reporting to authority were less consistent and depended on grade level. For primary school students, but not secondary school students, there were consistent within-person carry-over effects for both assertive defending and reporting to authority. These findings are consistent with previous research which has found that younger children are generally more likely to defend their victimized peers (Ma et al., 2019). Although there was no a priori hypothesis about this finding, it is possible that, compared to younger students, older students may not consistently use assertive defending or reporting to authority, but instead their use of these strategies may depend more on the specific incident (e.g., characteristics of the victimized student and perpetrator) or contextual factors (e.g., classroom context). Indeed, in adolescence, the social environment is more salient and, consistent with theoretical models of Prosocial Risk Taking, adolescents may weigh the potential social costs or risks of defending more heavily compared to younger children (Do et al., 2017). The potential costs may be particularly salient for assertive defending, which would involve publicly challenging a (potentially powerful) peer, and reporting to authority, which could signal to their peers a lack of autonomy. Thus, the use of these defending types may be less consistent in adolescence compared to younger children. Given these findings, future anti-bullying interventions should carefully consider the different types of defending when encouraging adolescents to defend, as they may be more receptive to engaging in some types of defending than others.

Across the full sample, there was no significant within-person concurrent association between defending and victimization at any time point. However, when considering possible grade level differences, (some) support was found for a positive within-person association between assertive defending and self-reported victimization (at T1) and between reporting to authority and peer-reported victimization (at T2) among secondary students only. This suggests that, for older students, elevated levels of defending (compared to usual levels) may be concurrently associated with higher levels of victimization (compared to usual levels). Although these findings were not robust across all waves, it is consistent with previous research demonstrating that a positive, concurrent association between defending and victimization was more likely for older youth compared to younger children (Ma et al., 2019).

However, the main focus of the current study was to examine the *prospective* within-person associations between defending and victimization. When testing these bidirectional associations over time, the findings did not indicate that (any type of) defending behavior was positively associated with subsequent (self- or peer-reported) victimization on the within-person level, or vice versa. Thus, no evidence was found that defending put youth at risk for subsequent victimization, or that previous victimization experiences increased the likelihood that youth would defend. This pattern was consistent across informants of victimization, types of defending, gender, and grade levels.

There are several possible explanations for why defending might not put youth at risk for subsequent victimization. Youth who choose to defend their peers may do so because they have social resources that protect them from possible retaliation. Defending (particularly assertive defending) may itself be a signal of social power that subsequently avoids increasing risk of retaliation. There is some evidence that perpetrators tend to bully who they perceive as an “easy” target, such as peers with little social support (Serdiouk et al., 2013), and youth’s willingness to defend others may keep others from viewing them as an “easy” next target. Given that anti-bullying interventions often encourage defending, the overall (lack of) evidence for defending as a risk factor for victimization suggests that there is no clear need to modify these programs to avoid iatrogenic effects (i.e., defending leading to victimization).

However, although this study can be considered a robust test of the bidirectional, within-person associations between defending and victimization, there are several factors that should still be considered by future research. First, even if defending and victimization are generally not predictors of one another, it is still possible that their prospective association depends on other factors (e.g., social status, empathy). Although previous research examining the between-person associations of defending on subsequent victimization did not find that popularity moderated this link (Malamut et al., 2023), a different pattern of findings might have emerged when considering within-person associations. In addition, although this study focused on both self- and peer-reports of actual victimization experiences in relation to defending, another important factor may be adolescents’ fear of victimization. Insofar as youth may be reluctant to defend their victimized peers out of fear of becoming victimized themselves (Strindberg et al., 2020), this fear can exist regardless of whether defending actually tends to result in increased victimization experiences.

### Strengths, Limitations and Future Directions

The strengths of this study include a multi-informant, longitudinal design and an analytic approach that teases apart between- and within-person associations. In addition, different types of defending were considered, rather than treating defending as a homogenous behavior. However, there were also a number of limitations. Even though it was a strength to include different types of defending, the different defending behaviors were strongly correlated in this study, which caused convergence issues and may have limited the ability to find patterns that are unique to each type. In addition, the overall rates of victimization were relatively low- still, the rates of victimization in this sample are consistent with other research.

Although both gender and grade level were considered as possible moderators, there are other factors that were not accounted for in the current study due to model complexity. For example, whether or not there are within-person associations between defending and victimization may be moderated by other individual characteristics of the adolescents that are known to be important risk factors for victimization (e.g., status, internalizing problems) or their relationship (e.g., whether they are friends with the victimized student). It is also possible that aspects of the classroom could impact these associations. For example, in classroom contexts that promote bullying (e.g., where bullying is rewarded with popularity, or where pro-bullying attitudes prevail), it may be more likely that defending could lead to within-person increases in victimization.

The analytic approach used allowed us to disentangle between- and within-person associations; however, other analytic approaches should also be considered to investigate this important question. For example, analyses based on person-centered trajectories could be useful to investigate potential heterogeneity among youth who defend and how differences among these youth relate to trajectories of victimization over time (and vice versa).

Lastly, as with all longitudinal studies, it is important to consider the timing between waves. First, it is important to note that the data collection for this study overlapped with the COVID-19 pandemic. However, the data collection was not disrupted by remote schooling as Finnish schools maintained in-person instruction after a two-month school closure in the spring of 2020. In addition, given that the study spanned two academic years, the timeframe from T2 to T3 slightly differed from the timeframe from T1 to T2 and T3 to T4. It is also possible that the timing of the waves in the current study was not ideal for all paths of interest. For example, previous victimization may be more likely to be positively associated with defending with more distance in time – perhaps when their own victimization is no longer on-going. Future research investigating whether victimization is positively associated with defending over time should differentiate between those who have previously been victimized (but no longer are) and those who are still experiencing victimization.

## Conclusion

Given that anti-bullying interventions often include directly or indirectly encouraging bystanders to defend their victimized peers (Salmivalli et al., 2021), it is critical to rigorously investigate potential risks for peer bystanders – to ensure that these interventions will not inadvertently cause harm. The current study did not find any support that defending was positively associated with victimization on the within-person level (or between-person level), and this was consistent across three types of defending (comforting defending, assertive defending, reporting to authority) and two informants of victimization (self and peers). Although this offers preliminary good news for anti-bullying interventions, future research is encouraged to examine other factors that may impact whether defending puts youth at risk for victimization.

## Supplementary information

Supplementary Information

## Data Availability

Fully anonymized data and analysis code are available from the first author upon reasonable request.
