# Supplementary material for: Defending Behavior and Victimization: Between- and Within-Person Associations
Source: J Youth Adolesc. 2025 Mar 20;54(7):1646–58. doi: 10.1007/s10964-025-02168-x (PMC12245965; doi:10.1007/s10964-025-02168-x)
Supplement: Supplementary file 1 — Supplementary Information [file 10964_2025_2168_MOESM1_ESM.docx]

Manuscript: Defending Behavior and Victimization: Between- and Within-Person Associations
Authors: Sarah T. Malamut, Claire F. Garandeau, & Christina Salmivalli

| Table S1  *Comforting defending and self-reported victimization by grade level (unconstrained RI-CLPM)* | | | |
| --- | --- | --- | --- |
|  | Primary school (n = 1713) |  | Secondary school (n = 3136) |
|  | Beta (95% CI) |  | Beta (95% CI) |
| **Lagged (autoregressive) effects** |  |  |  |
| Defending T2 on defending T1 | .39 (.10,.68)^a^ |  | .23 (-.00,.47)^a^ |
| Defending T3 on defending T2 | .46 (.23,.68)^a^ |  | .15 (-.25,.55)^a^ |
| Defending T4 on defending T3 | **.51 (.34,.69)^a^** |  | **.09 (-.28,.47)^b^** |
| Victimization T2 on victimization T1 | .29 (.12,.47)^a^ |  | .20 (.00,.40)^a^ |
| Victimization T3 on victimization T2 | .34 (.11,.58)^a^ |  | .45 (.27,.62)^a^ |
| Victimization T4 on victimization T3 | .14 (-.14,.43)^a^ |  | .35 (.17,.53)^a^ |
| **Cross-lagged effects** |  |  |  |
| Victimization T2 on defending T1 | .03 (-.10,.15)^a^ |  | .08 (-.01,.17)^a^ |
| Victimization T3 on defending T2 | -.06 (-.18,.05)^a^ |  | -.00 (-.08,.08)^a^ |
| Victimization T4 on defending T3 | -.01 (-.13,.11)^a^ |  | -.05 (-.11,.02)^a^ |
| Defending T2 on victimization T1 | -.10 (-.22, .02)^a^ |  | .02 (-.08,.13)^b^ |
| Defending T3 on victimization T2 | -.02 (-.08,.05)^a^ |  | .02 (-.11,.15)^a^ |
| Defending T4 on victimization T3 | -.06 (-.16,.03)^a^ |  | .02 (-.07,.11)^a^ |
| **Within-person covariances** |  |  |  |
| Defending T1 with victimization T1 | -.06 (-.16,.05)^a^ |  | .08 (-.02,.17)^b^ |
| Defending T2 with victimization T2 | -.06 (-.17,.04)^a^ |  | .08 (-.06,.22)^b^ |
| Defending T3 with victimization T3 | -.01 (-.10,.09)^a^ |  | -.03 (-.10,.04)^a^ |
| Defending T4 with victimization T4 | .03 (-.08,.13)^a^ |  | -.05 (-.11,.01)^a^ |
| **Between-person covariances** |  |  |  |
| Defending with victimization | -.11 (-.30,.07)^a^ |  | -.10 (-.23,.02)^a^ |
| **Controlling for gender** |  |  |  |
| Defending T1 on girl | .38 (.31, .45)^a^ |  | .33 (.27,.38)^a^ |
| Defending T2 on girl | **.38 (.32, .44)^a^** |  | **.27 (.22,.31)^b^** |
| Defending T3 on girl | **.38 (.32, .43)^a^** |  | **.29 (.25,.33)^b^** |
| Defending T4 on girl | .25 (.18, .32)^a^ |  | .22 (.18,.26)^a^ |
| Victimization T1 on girl | -.05 (-.10, .01)^a^ |  | .01 (-.04,.06)^a^ |
| Victimization T2 on girl | .02 (-.03, .06)^a^ |  | .01 (-.04,.06)^a^ |
| Victimization T3 on girl | .02 (-.05, .08)^a^ |  | .05 (-.00,.09)^a^ |
| Victimization T4 on girl | .06 (.01, .11)^a^ |  | .02 (-.04,.08)^a^ |
| *Note.* Standardized estimates are presented. Different superscripts imply significant differences in parameters between primary students and secondary students based on overlapping confidence intervals. The bolded parameters indicate a significant grade level difference where the effect was significant for one or both groups. When controlling for gender, the reference group was boy. | | | |

| Table S2  *Assertive defending and self-reported victimization by grade level (unconstrained RI-CLPM)* | | | |
| --- | --- | --- | --- |
|  | Primary school (n = 1713) |  | Secondary school (n = 3136) |
|  | Beta (95% CI) |  | Beta (95% CI) |
| **Lagged (autoregressive) effects** |  |  |  |
| Defending T2 on defending T1 | .29 (-.06,.63)^a^ |  | -.24 (-.73,.24)^b^ |
| Defending T3 on defending T2 | **.46 (.20,.71)^a^** |  | **-.43 (-1.29,.42)^b^** |
| Defending T4 on defending T3 | **.49 (.28,.69)^a^** |  | **.19 (-.05,.43)^b^** |
| Victimization T2 on victimization T1 | .29 (.12,.47)^a^ |  | .21 (.01,.41)^a^ |
| Victimization T3 on victimization T2 | .35 (.12,.58)^a^ |  | .44 (.26,.62)^a^ |
| Victimization T4 on victimization T3 | .15 (-.14,.43)^a^ |  | .36 (.18,.54)^a^ |
| **Cross-lagged effects** |  |  |  |
| Victimization T2 on defending T1 | .05 (-.07,.17)^a^ |  | .08 (-.02,.17)^a^ |
| Victimization T3 on defending T2 | -.07 (-.18,.03)^a^ |  | .06 (-.03,.16)^b^ |
| Victimization T4 on defending T3 | .01 (-.12,.15)^a^ |  | -.04 (-.11,.03)^a^ |
| Defending T2 on victimization T1 | -.06 (-.17, .06)^a^ |  | .08 (-.05,.21)^b^ |
| Defending T3 on victimization T2 | -.08 (-.15,-.02)^a^ |  | .10 (-.10,.30)^a^ |
| Defending T4 on victimization T3 | -.07 (-.18,.04)^a^ |  | .01 (-.08,.10)^a^ |
| **Within-person covariances** |  |  |  |
| Defending T1 with victimization T1 | **-.03 (-.12,.07)^a^** |  | **.10 (.01,.19)^b^** |
| Defending T2 with victimization T2 | .00 (-.11,.11)^a^ |  | .18 (-.05,.40)^a^ |
| Defending T3 with victimization T3 | -.04 (-.12,.03)^a^ |  | .02 (-.08,.13)^a^ |
| Defending T4 with victimization T4 | -.02 (-.13,.10)^a^ |  | -.02 (-.06,.02)^a^ |
| **Between-person covariances** |  |  |  |
| Defending with victimization | -.07 (-.24,.11)^a^ |  | -.06 (-.18,.05)^a^ |
| **Controlling for gender** |  |  |  |
| Defending T1 on girl | .28 (.21, .35)^a^ |  | .21 (.16,.27)^a^ |
| Defending T2 on girl | .22 (.16, .29)^a^ |  | .16 (.12,.21)^a^ |
| Defending T3 on girl | .20 (.15, .26)^a^ |  | .17 (.13,.21)^a^ |
| Defending T4 on girl | .17 (.11, .23)^a^ |  | .15 (.10,.20)^a^ |
| Victimization T1 on girl | -.05 (-.11, .01)^a^ |  | .01 (-.04,.07)^b^ |
| Victimization T2 on girl | .01 (-.04, .06)^a^ |  | .01 (-.03,.06)^a^ |
| Victimization T3 on girl | .02 (-.04, .09)^a^ |  | .05 (.00,.10)^a^ |
| Victimization T4 on girl | .06 (.00, .11)^a^ |  | .02 (-.04,.08)^a^ |
| *Note.* Standardized estimates are presented. Different superscripts imply significant differences in parameters between primary students and secondary students based on overlapping confidence intervals. The bolded parameters indicate a significant grade level difference where the effect was significant for one or both groups. When controlling for gender, the reference group was boy. | | | |

| Table S3  *Assertive defending and peer-reported victimization by grade level (unconstrained RI-CLPM)* | | | |
| --- | --- | --- | --- |
|  | Primary school (n = 1704) |  | Secondary school (n = 3070) |
|  | Beta (95% CI) |  | Beta (95% CI) |
| **Lagged (autoregressive) effects** |  |  |  |
| Defending T2 on defending T1 | .28 (-.07,.63)^a^ |  | -.23 (-.73,.27)^b^ |
| Defending T3 on defending T2 | **.45 (.19,.71)^a^** |  | **-.49 (-1.34,.36)^b^** |
| Defending T4 on defending T3 | **.49 (.28,.69)^a^** |  | **.15 (-.12,.41)^b^** |
| Victimization T2 on victimization T1 | .33 (.05,.60)^a^ |  | .36 (.16,.56)^a^ |
| Victimization T3 on victimization T2 | .43 (.13,.73)^a^ |  | .43 (.20,.66)^a^ |
| Victimization T4 on victimization T3 | .48 (.11,.84)^a^ |  | .25 (-.04,.53)^a^ |
| **Cross-lagged effects** |  |  |  |
| Victimization T2 on defending T1 | .12 (-.09,.33)^a^ |  | .02 (-.13,.17)^a^ |
| Victimization T3 on defending T2 | -.06 (-.23,.12)^a^ |  | .01 (-.16,.18)^a^ |
| Victimization T4 on defending T3 | -.00 (-.10,.10)^a^ |  | .05 (-.08,.18)^a^ |
| Defending T2 on victimization T1 | -.03 (-.25, .19)^a^ |  | .10 (-.16,.36)^a^ |
| Defending T3 on victimization T2 | -.04 (-.14,.07)^a^ |  | .29 (-.07,.66)^a^ |
| Defending T4 on victimization T3 | -.04 (-.21,.14)^a^ |  | .17 (-.02,.36)^b^ |
| **Within-person covariances** |  |  |  |
| Defending T1 with victimization T1 | .16 (-.09,.40)^a^ |  | .09 (-.09,.26)^a^ |
| Defending T2 with victimization T2 | .02 (-.12,.15)^a^ |  | .24 (-.07,.54)^a^ |
| Defending T3 with victimization T3 | .01 (-.09,.11)^a^ |  | .19 (-.03,.41)^a^ |
| Defending T4 with victimization T4 | .07 (-.07,.21)^a^ |  | .09 (-.07,.25)^a^ |
| **Between-person covariances** |  |  |  |
| Defending with victimization | -.07 (-.29,.15)^a^ |  | -.08 (-.23,.07)^a^ |
| **Controlling for gender** |  |  |  |
| Defending T1 on girl | .28 (.21, .34)^a^ |  | .21 (.16,.27)^a^ |
| Defending T2 on girl | .22 (.16, .29)^a^ |  | .17 (.12,.22)^a^ |
| Defending T3 on girl | .20 (.14, .26)^a^ |  | .17 (.13,.22)^a^ |
| Defending T4 on girl | .16 (.11, .22)^a^ |  | .15 (.10,.20)^a^ |
| Victimization T1 on girl | -.10 (-.17, .02)^a^ |  | -.03 (-.09,.02)^a^ |
| Victimization T2 on girl | **-.05 (-.12, .03)^a^** |  | **-.12 (-.18,-.06)^b^** |
| Victimization T3 on girl | -.03 (-.09, .03)^a^ |  | -.05 (-.10,-.00)^a^ |
| Victimization T4 on girl | -.04 (-.10, .02)^a^ |  | -.03 (-.07,.02)^a^ |
| *Note.* Standardized estimates are presented. Different superscripts imply significant differences in parameters between primary students and secondary students based on overlapping confidence intervals. The bolded parameters indicate a significant grade level difference where the effect was significant for one or both groups. When controlling for gender, the reference group was boy. | | | |

| Table S4  *Reporting to authority and self-reported victimization by grade level (unconstrained RI-CLPM)* | | | |
| --- | --- | --- | --- |
|  | Primary school (n = 1713) |  | Secondary school (n = 3136) |
|  | Beta (95% CI) |  | Beta (95% CI) |
| **Lagged (autoregressive) effects** |  |  |  |
| Defending T2 on defending T1 | .29 (-.07,.64)^a^ |  | -.24 (-.52,.04)^b^ |
| Defending T3 on defending T2 | **.49 (.22,.76)^a^** |  | **-.13 (-.63,.36)^b^** |
| Defending T4 on defending T3 | **.52 (.32,.72)^a^** |  | **-.03 (-.42,.37)^b^** |
| Victimization T2 on victimization T1 | .29 (.11,.47)^a^ |  | .22 (.02,.42)^a^ |
| Victimization T3 on victimization T2 | .34 (.11,.58)^a^ |  | .45 (.28,.62)^a^ |
| Victimization T4 on victimization T3 | .14 (-.14,.43)^a^ |  | .36 (.18,.54)^a^ |
| **Cross-lagged effects** |  |  |  |
| Victimization T2 on defending T1 | .05 (-.07,.17)^a^ |  | .04 (-.04,.11)^a^ |
| Victimization T3 on defending T2 | -.03 (-.15,.09)^a^ |  | .02 (-.04,.08)^a^ |
| Victimization T4 on defending T3 | -.00 (-.11,.11)^a^ |  | -.02 (-.08,.04)^a^ |
| Defending T2 on victimization T1 | -.02 (-.17, .12)^a^ |  | .07 (-.10,.23)^a^ |
| Defending T3 on victimization T2 | -.03 (-.09,.02)^a^ |  | .04 (-.08,.16)^a^ |
| Defending T4 on victimization T3 | -.05 (-.13,.04)^a^ |  | -.02 (-.13,.10)^a^ |
| **Within-person covariances** |  |  |  |
| Defending T1 with victimization T1 | .02 (-.08,.13)^a^ |  | .08 (.01,.16)^a^ |
| Defending T2 with victimization T2 | -.04 (-.15,.08)^a^ |  | .13 (-.11,.37)^a^ |
| Defending T3 with victimization T3 | -.01 (-.10,.09)^a^ |  | .05 (-.02,.12)^a^ |
| Defending T4 with victimization T4 | .01 (-.10,.12)^a^ |  | -.00 (-.06,.05)^a^ |
| **Between-person covariances** |  |  |  |
| Defending with victimization | -.10 (-.31,.11)^a^ |  | -.06 (-.18,.06)^a^ |
| **Controlling for gender** |  |  |  |
| Defending T1 on girl | **.31 (.24, .39)^a^** |  | **.18 (.12,.24)^b^** |
| Defending T2 on girl | **.32 (.26, .38)^a^** |  | **.17 (.12,.21)^b^** |
| Defending T3 on girl | .21 (.15, .27)^a^ |  | .21 (.16,.25)^a^ |
| Defending T4 on girl | .17 (.11, .24)^a^ |  | .12 (.08,.17)^a^ |
| Victimization T1 on girl | -.05 (-.10, .01)^a^ |  | .02 (-.04,.08)^b^ |
| Victimization T2 on girl | .02 (-.03, .06)^a^ |  | .01 (-.03,.06)^a^ |
| Victimization T3 on girl | .02 (-.05, .08)^a^ |  | .05 (.01,.10)^a^ |
| Victimization T4 on girl | .06 (.01, .11)^a^ |  | .02 (-.04,.08)^a^ |
| *Note.* Standardized estimates are presented. Different superscripts imply significant differences in parameters between primary students and secondary students based on overlapping confidence intervals. The bolded parameters indicate a significant grade level difference where the effect was significant for one or both groups. When controlling for gender, the reference group was boy. | | | |

| Table S5  *Reporting to authority and peer-reported victimization by grade level (unconstrained RI-CLPM)* | | | |
| --- | --- | --- | --- |
|  | Primary school (n = 1704) |  | Secondary school (n = 3070) |
|  | Beta (95% CI) |  | Beta (95% CI) |
| **Lagged (autoregressive) effects** |  |  |  |
| Defending T2 on defending T1 | .27 (-.06,.59)^a^ |  | -.22 (-.48,.03)^b^ |
| Defending T3 on defending T2 | **.48 (.24,.73)^a^** |  | **-.17 (-.66,.32)^b^** |
| Defending T4 on defending T3 | **.52 (.33,.70)^a^** |  | **-.07 (-.47,.33)^b^** |
| Victimization T2 on victimization T1 | .34 (.07,.61)^a^ |  | .37 (.17,.57)^a^ |
| Victimization T3 on victimization T2 | .43 (.13,.73)^a^ |  | .46 (.24,.67)^a^ |
| Victimization T4 on victimization T3 | .48 (.12,.83)^a^ |  | .26 (-.02,.54)^a^ |
| **Cross-lagged effects** |  |  |  |
| Victimization T2 on defending T1 | .08 (-.10,.25)^a^ |  | -.00 (-.10,.10)^a^ |
| Victimization T3 on defending T2 | -.13 (-.29,.03)^a^ |  | -.09 (-.24,.06)^a^ |
| Victimization T4 on defending T3 | .05 (-.06,.16)^a^ |  | .02 (-.09,.12)^a^ |
| Defending T2 on victimization T1 | .07 (-.15, .29)^a^ |  | -.01 (-.18,.16)^a^ |
| Defending T3 on victimization T2 | .08 (.01,.16)^a^ |  | .22 (-.01,.44)^a^ |
| Defending T4 on victimization T3 | -.02 (-.19,.16)^a^ |  | .18 (-.03,.40)^a^ |
| **Within-person covariances** |  |  |  |
| Defending T1 with victimization T1 | .10 (-.13,.34)^a^ |  | -.03 (-.15,.10)^a^ |
| Defending T2 with victimization T2 | **-.07 (-.21,.07)^a^** |  | **.25 (.04,.45)^b^** |
| Defending T3 with victimization T3 | -.03 (-.16,.11)^a^ |  | .11 (-.05,.28)^a^ |
| Defending T4 with victimization T4 | .07 (-.09,.23)^a^ |  | .14 (.01,.26)^a^ |
| **Between-person covariances** |  |  |  |
| Defending with victimization | -.03 (-.24,.18)^a^ |  | -.01 (-.13,.11)^a^ |
| **Controlling for gender** |  |  |  |
| Defending T1 on girl | **.32 (.24,.39)^a^** |  | **.19 (.13,.25)^b^** |
| Defending T2 on girl | **.32 (.26,.38)^a^** |  | **.17 (.12,.21)^b^** |
| Defending T3 on girl | .21 (.15,.28)^a^ |  | .20 (.16,.25)^a^ |
| Defending T4 on girl | .17 (.11,.24)^a^ |  | .12 (.08,.17)^a^ |
| Victimization T1 on girl | -.10 (-.17,-.02)^a^ |  | -.03 (-.09,.02)^b^ |
| Victimization T2 on girl | -.05 (-.12,.02)^a^ |  | -.12 (-.17,-.06)^a^ |
| Victimization T3 on girl | -.03 (-.08,.03)^a^ |  | -.05 (-.10,-.00)^a^ |
| Victimization T4 on girl | -.04 (-.10,.02)^a^ |  | -.02 (-.07,.02)^a^ |
| *Note.* Standardized estimates are presented. Different superscripts imply significant differences in parameters between primary students and secondary students based on overlapping confidence intervals. The bolded parameters indicate a significant grade level difference where the effect was significant for one or both groups. When controlling for gender, the reference group was boy. | | | |
